# Phytochemical Composition, Elemental Profiles, Antioxidant Activity, and Xenobiotic Relevance of Rosehip and Sea Buckthorn Fruits from Armenia

**DOI:** 10.3390/jox16060202

**Published:** 2026-10-21

**Authors:** Lusine Sahakyan, Avetis Tsaturyan, Ela Minasyan, Lusine Hayrapetyan, Ani Chakhoyan, Valodya Mirzoyan, Narine Zakaryan, Rosanna Palumbo, Caterina Vicidomini, Giovanni N. Roviello, Ashot Saghyan

**Affiliations:** 1 Institute of Pharmacy, Yerevan State University, Yerevan 0025, Armenia; lusine_sahakyan@ysu.am (L.S.); saghyan@ysu.am (A.S.); 2 Scientific and Production Center “Armbiotechnology”, National Academy of Science, Yerevan 0056, Armenia; 3 Faculty of Biology, Yerevan State University, Yerevan 0025, Armenia; 4 Institute of Biostructures and Bioimaging (IBB), National Research Council of Italy (CNR), 80145 Naples, Italy

**Keywords:** vitamin C, soluble sugars, ICP-OES, HPLC, DPPH assay, potentially toxic elements, rosehip, buckthorn

## Abstract

Medicinal and edible plants are valuable sources of biologically active compounds whose composition may vary with botanical identity, geographical origin, environmental conditions, and post-harvest processing. In addition to essential mineral constituents, such plants may accumulate non-essential and potentially toxic elements relevant to exogenous chemical exposure and xenobiotic research. This study characterized the phytochemical composition, essential and non-essential elemental profile, and antioxidant activity of rosehip (*Rosa* spp.) and sea buckthorn (*Hippophae rhamnoides* L.) fruits collected from different locations in Armenia. Soluble sugars and vitamin C were determined by HPLC, elemental composition by ICP-OES, and radical-scavenging activity by the DPPH assay. In fresh rosehip, vitamin C ranged from 130 to 900 mg/100 g, compared with 11.9–40.0 mg/100 g in sea buckthorn. Potassium was predominant in both fruits. In the analyzed samples, the measured Ca and Mg concentrations were numerically higher in rosehip, whereas higher Fe concentrations were observed in sea buckthorn. Potentially toxic elements, including As, Cd, and Pb, were detected at different concentrations among the analyzed samples. In the selected paired rosehip samples, lower DPPH radical-scavenging activity was measured in the shade-dried materials than in the corresponding fresh materials, while the analyzed sea buckthorn samples showed high DPPH radical-scavenging activity under the applied assay conditions. These findings provide a comparative characterization of the phytochemical and elemental profiles of the investigated fruits and highlight the xenobiotic relevance of the detected non-essential elements.

## 1. Introduction

Medicinal and edible plants are complex biological matrices containing numerous low-molecular-weight constituents that may contribute to nutritional value, pharmacological activity, and potential health effects. Their chemical composition is influenced by genetic background, environmental conditions, developmental stage, and post-harvest processing, resulting in substantial variability in phytochemical and elemental profiles [1,2,3]. In the context of xenobiotic assessment, a xenobiotic is generally defined as a compound foreign to a living organism [2]. Naturally occurring plant bioactive compounds are therefore distinguished here from xenobiotics in the strict sense when considered as intrinsic constituents of the plant matrix. However, natural products can serve as structural leads for chemical modification and optimization in drug discovery, generating derivatives with altered or enhanced pharmaceutical properties. In addition, medicinal and edible plants may contain non-essential and potentially toxic elements, including As, Ba, Cd, and Pb, providing a more direct link between plant materials, exogenous chemical exposure, and xenobiotic assessment [3]. Consequently, integrated characterization of both phytochemical constituents and essential and non-essential elements is important for evaluating the quality, biological properties, and xenobiotic relevance of medicinal and edible plant materials.

Rosehips, the pseudofruits of *Rosa* spp. (Rosaceae), are widely consumed and used as raw materials for traditional preparations, functional foods, and phytoproducts. They contain soluble carbohydrates, organic acids, phenolic compounds, flavonoids, carotenoids, tocopherols, and mineral elements and are particularly recognized for their high vitamin C content [4,5,6,7,8,9]. Rosehip fruits also contain terpenoid constituents; triterpenic acids such as ursolic, oleanolic, and betulinic acids have been identified in *Rosa canina* preparations [10]. These compounds further expand the phytochemical diversity of rosehip and have been investigated for their potential pharmacological activities [11]. However, considerable compositional variability has been reported among *Rosa* species, cultivars, geographical origins, and stages of fruit maturity [4,5,6,7,9]. Mineral profiling has likewise demonstrated substantial differences in K, Ca, Mg, Fe, Mn, Zn, and other elements among *Rosa* fruits [4,12,13]. These observations indicate that rosehip represents a chemically heterogeneous plant material and emphasize the importance of botanical authentication and sampling origin in comparative studies.

Rosehip fruits are also characterized by substantial antioxidant potential. Their radical-scavenging properties have been associated with the combined contribution of ascorbic acid, phenolic compounds, flavonoids, carotenoids, and other redox-active constituents [6,8,14,15]. Accordingly, antioxidant activity does not necessarily parallel the concentration of a single phytochemical marker. Post-harvest processing may further modify this profile, as drying can affect the stability and extractability of vitamin C and other antioxidant constituents [16]. Thus, simultaneous evaluation of chemical composition and antioxidant activity provides a more comprehensive characterization than assessment based on an individual constituent alone.

Sea buckthorn (*Hippophae rhamnoides* L., Elaeagnaceae) is another medicinal and edible fruit-bearing plant valued for its diverse phytochemical composition. Its berries contain vitamins, carotenoids, polyphenols, flavonoids, fatty acids, phytosterols, soluble carbohydrates, organic acids, and mineral elements [17,18]. The phytochemical diversity of *H. rhamnoides* also includes terpenoid constituents; triterpenic acids such as oleanolic acid, ursolic acid, and 19α-hydroxyursolic acid have been isolated directly from its fruits [19]. Alkaloids have also been reported among the broader classes of specialized metabolites identified in *H. rhamnoides* [20]. These constituents contribute to the nutritional and antioxidant properties of sea buckthorn and have supported its increasing use in foods and phytoproducts [17,18]. As in rosehip, its chemical composition is highly variable. Changes in sugars, ascorbic acid, phenolic compounds, and antioxidant activity have been reported among genotypes, geographical origins, and different stages of fruit development and ripening [21,22,23]. Such variability demonstrates the importance of region-specific characterization rather than direct extrapolation of compositional data obtained from other geographical populations.

The chemical variability of wild fruits may reflect complex interactions between plant genotype and environmental conditions, including climate, altitude, soil properties, and water availability [2,3,12,13]. Because these factors interact, differences among naturally occurring populations are more appropriately considered in terms of eco-geographical variability unless individual environmental variables are independently investigated. Armenia, with its pronounced topographic and ecological heterogeneity, represents an interesting setting for such studies. Wild edible and medicinal plants remain important components of Armenian food and ethnomedicinal traditions, as demonstrated by ethnobotanical investigations documenting their extensive use and commercialization [24]. Nevertheless, analytical information on rosehip and sea buckthorn fruits originating from different Armenian locations remains limited. To the best of our knowledge, an integrated comparison combining botanical authentication, soluble sugar and vitamin C profiling, multi-element analysis, and radical-scavenging activity of Armenian rosehip and sea buckthorn fruits has not previously been reported. Moreover, the distinction between essential and non-essential elements has received limited attention in comparative studies of these fruits, despite its relevance to the assessment of elemental quality and potential xenobiotic exposure. However, the chemical composition of wild fruits may be influenced by multiple interacting factors, including botanical identity, environmental conditions, developmental stage, and post-harvest processing; therefore, differences among samples collected from different locations cannot necessarily be attributed to geographical origin alone.

Therefore, the present study aimed to comparatively characterize the phytochemical composition, essential and non-essential elemental profiles, and antioxidant activity of rosehip (*Rosa* spp.) and sea buckthorn (*Hippophae rhamnoides* L.) fruits collected from different locations in Armenia. Particular attention was given to non-essential and potentially toxic elements because of their relevance to exogenous chemical exposure and xenobiotic research.

## 2. Materials and Methods

### 2.1. Chemicals and Reagents

HPLC-grade acetonitrile (99.9%, Sigma-Aldrich, St. Louis, MO, USA) was used as the mobile phase for chromatographic analyses. Deionized Milli-Q water (Millipore, Bedford, MA, USA) was used for the preparation of all aqueous solutions and sample extraction. Metaphosphoric acid (analytical grade, Reakhim, Moscow, Russia) was used for the extraction and stabilization of vitamin C. The stable free radical 2,2-diphenyl-1-picrylhydrazyl (DPPH) was purchased from Merck (Darmstadt, Germany). Ethanol (96%, Lizin OJSC, Yerevan, Armenia) was used for the preparation of plant extracts for antioxidant activity determination. Analytical reference standards of L-ascorbic acid, D-fructose, D-glucose, and sucrose were obtained from Merck (Darmstadt, Germany) and Sigma-Aldrich (St. Louis, MO, USA). For elemental analysis, concentrated nitric acid (69%, trace analysis grade) was used for microwave-assisted acid digestion. Calibration of the ICP-OES system was performed using a certified multi-element standard solution containing 25 elements (Product No. 9308543, PerkinElmer Reagents, Shelton, CT, USA), each at a concentration of 100 mg L^−1^ in a 5% nitric acid matrix. Unless otherwise stated, all chemicals and reagents used in this study were of analytical or HPLC grade.

### 2.2. Plant Material, Sample Collection and Botanical Identification

Fruit samples of rosehip (*Rosa* spp.) and sea buckthorn (*H. rhamnoides* L.) were collected from naturally growing populations in different locations in Armenia during two consecutive harvesting seasons. Sea buckthorn fruits were collected in October 2024 from four sampling sites located in the Gegharkunik Province, including the northeastern shoreline of Lake Sevan (Drakhtik area) and the Sevan Basin. Rosehip fruits were collected during September-October 2025 from seven sampling sites distributed across the Kotayk, Gegharkunik, Tavush, and Aragatsotn Provinces. Detailed information on the sampling sites, geographical coordinates, species identification, collection year, and sample codes is presented in Table 1.

Immediately after collection, the fruits were manually cleaned to remove foreign materials and mechanical impurities. For the selected rosehip samples used for fresh-versus-dried comparisons, fruits were arranged in a single layer and shade-dried under ambient laboratory conditions in a well-ventilated room, protected from direct sunlight, for approximately 20–30 days until constant mass was achieved. Ambient temperature and relative humidity were not specifically controlled or recorded during the drying period. The final moisture content of the shade-dried samples was below 7%, as determined gravimetrically to constant mass. Vitamin C and soluble sugar concentrations in fresh fruits were expressed per 100 g of fresh material, whereas the corresponding values for shade-dried fruits were expressed per 100 g of dried material. Because the fresh-fruit results were not converted to a dry-weight basis, direct quantitative assessment of vitamin C retention or loss during shade-drying was not performed. Fresh samples were stored at −18 °C until phytochemical analyses. Shade-dried samples intended for elemental analysis were stored in a dry, dark environment until analysis.

All seven fresh rosehip samples were analyzed for soluble sugars and vitamin C. Corresponding shade-dried materials were additionally analyzed for three rosehip samples (RC-4, Sevan; RC-6, Goght; and RC-7, Aparan), based on the availability of paired fresh and shade-dried material rather than on predefined selection criteria. All four fresh sea buckthorn samples were analyzed for soluble sugars and vitamin C. Elemental profiling by inductively coupled plasma optical emission spectrometry (ICP-OES) was performed using all shade-dried rosehip (seven samples) and sea buckthorn (four samples) fruit samples. Botanical identification of all collected plant materials was performed by taxonomists from the Department of Botany and Mycology, Faculty of Biology, Yerevan State University (Yerevan, Armenia). Representative voucher specimens were prepared and deposited in the Herbarium of Yerevan State University (ERCB). All sea buckthorn samples were identified as *Hippophae rhamnoides* L., the only naturally occurring species of the genus *Hippophae* in Armenia. Representative voucher specimens collected from the Sevan Basin and Drakhtik localities were deposited in the Herbarium of Yerevan State University under voucher numbers ERCB 13912 and ERCB 13866, respectively. Botanical examination revealed that most of the investigated rosehip samples belonged to *Rosa canina* L. The sample collected from Geghashen was identified as *Rosa corymbifera* Borkh. (voucher specimen ERCB 13923), whereas the sample collected from Sevqar was identified as *Rosa nisami* Sosn. (voucher specimen ERCB 13924). Representative voucher specimens of the investigated *Rosa* taxa were deposited in the Herbarium of Yerevan State University (ERCB). Photographs of representative plants and fruits, together with voucher specimens of the botanically identified taxa, are provided in the Supplementary Materials (Figure S1).

### 2.3. Determination of Soluble Sugars by HPLC-RID

Soluble sugars were determined by high-performance liquid chromatography with refractive index detection (HPLC-RID) using a Knauer Azura HPLC system (Knauer Scientific Instruments GmbH, Berlin, Germany) equipped with a refractive index detector (RID; Knauer Scientific Instruments GmbH, Berlin, Germany). Chromatographic separation was performed on an Asahipak NH_2_P-50 4D column (4.6 × 150 mm, 5 μm; Shodex, Showa Denko K.K., Tokyo, Japan). The mobile phase consisted of acetonitrile and deionized water (79:21, *v*/*v*), delivered under isocratic conditions at a flow rate of 1.0 mL min^−1^. The column temperature was maintained at 25 °C, the injection volume was 20 μL, and the chromatographic run time was 15 min.

Quantification of soluble sugars was performed using the external standard method with D-fructose, D-glucose, and sucrose as reference compounds. Five calibration levels, including a blank solution, were prepared for each analyte. The calibration ranges were 2.077–20.770 mg mL^−1^ for fructose, 2.683–26.830 mg mL^−1^ for glucose, and 2.450–24.500 mg mL^−1^ for sucrose. Each calibration level was analyzed in triplicate, and calibration curves were constructed by plotting peak area against analyte concentration. Linear regression equations and coefficients of determination (R^2^) were calculated for each sugar.

Prior to extraction, rosehip fruits were manually deseeded, and the pulp was ground using a high-speed universal disintegrator (FW80; Huanghua Faithful Instrument Co., Ltd., Huanghua, China) to obtain particles of approximately 1–2 mm. Sea buckthorn fruits were homogenized without seed removal. Approximately 1.0 g of each prepared sample was extracted with 20 mL of deionized water for 15 min at room temperature using a vortex mixer (SA8; Stuart, Bibby Scientific Ltd., Stone, Staffordshire, UK), followed by centrifugation at 3000 rpm for 10 min. The resulting supernatants were filtered through 0.20 μm membrane filters (E-Chrom Tech Co., Ltd., Taipei, Taiwan) and transferred into HPLC vials (Macherey-Nagel, Düren, Germany) prior to chromatographic analysis.

Individual sugars were identified by comparing their retention times with those of authentic reference standards analyzed under identical chromatographic conditions. Quantification was performed using the corresponding external calibration curves.

The analytical procedure was developed based on the general principles of the Official Methods of Analysis of AOAC INTERNATIONAL and optimized for the HPLC-RID determination of soluble sugars in rosehip and sea buckthorn fruits under the chromatographic conditions employed in this study [25,26].

### 2.4. Determination of Vitamin C by HPLC-DAD

Vitamin C content was determined by high-performance liquid chromatography with diode array detection (HPLC-DAD) following the method described by Medveckienė et al. [9,27], with minor modifications. Quantification was performed using the external standard method based on calibration curves prepared from L-ascorbic acid standard solutions. Chromatographic analysis was carried out using a Knauer Azura HPLC system (Knauer Scientific Instruments GmbH, Berlin, Germany) equipped with a diode array detector (DAD; Knauer Scientific Instruments GmbH, Berlin, Germany). Separation was achieved on a C18 column (250 × 4.6 mm, 5 μm; Thermo Scientific, Waltham, MA, USA). The mobile phase consisted of 0.1% metaphosphoric acid, delivered under isocratic conditions at a flow rate of 1.0 mL min^−1^. The column temperature was maintained at 25 °C, the injection volume was 10 μL, and chromatograms were recorded at 245 nm. Calibration curves were prepared using L-ascorbic acid standard solutions dissolved in 2% metaphosphoric acid. A reagent blank consisting of the extraction solvent was analyzed under identical chromatographic conditions.

Prior to extraction, rosehip fruits were manually deseeded, and the pulp was ground using a High-speed Universal Disintegrator FW80 to obtain particles of approximately 1–2 mm. Sea buckthorn fruits were homogenized without seed removal. Approximately 0.5 g of each sample was extracted with 2% metaphosphoric acid in a 10 mL volumetric flask. The extracts were vortex-mixed and incubated in the dark at room temperature for 20 min to ensure efficient extraction while minimizing oxidative degradation of ascorbic acid. The extracts were subsequently filtered through 0.20 μm membrane filters (E-Chrom Tech Co., Ltd., Taipei, Taiwan) and transferred into amber/orange colored HPLC vials for chromatographic analysis. Vitamin C was identified by comparing both the retention time and the UV absorption spectrum of the sample peak with those of an authentic L-ascorbic acid standard analyzed under identical chromatographic conditions and the results were expressed as mg per 100 g of analyzed sample. Quantification was performed using the corresponding external calibration curve. Each sample extract was analyzed three times as technical replicate measurements of the same prepared extract. These replicate measurements were used to assess analytical measurement repeatability and do not represent independent biological replicates.

### 2.5. Elemental Profiling by ICP-OES

Elemental profiling of dried fruit samples was performed using an Agilent 5800 VDV inductively coupled plasma optical emission spectrometer (ICP-OES; Agilent Technologies, Santa Clara, CA, USA) at the Analytical Testing Laboratory of the Educational Center for Drug Quality Control and Monitoring, Yerevan State University. Sample digestion was carried out using a Multiwave GO Plus microwave digestion system (Anton Paar GmbH, Graz, Austria). The digestion program consisted of a 15 min ramp to 200 °C followed by a 10 min hold at 200 °C. After cooling, the resulting clear digests were quantitatively transferred into 50 mL volumetric flasks, and the digestion vessels were rinsed with deionized water. The solutions were then diluted to volume with deionized water.

Elemental analysis was performed according to the procedure described in USP General Chapter <233> Elemental Impurities-Procedures [28]. The ICP-OES operating conditions were as follows: RF power, 1.2 kW; pump speed, 12 rpm; nebulizer gas flow, 0.7 L/min; plasma gas flow, 12 L/min; auxiliary gas flow, 1.0 L/min; borosilicate glass cyclonic double-pass spray chamber; radial viewing mode; sample uptake time, 25 s; stabilization time, 15 s; rinse time, 30 s; and read time, 5 s.

Elemental analysis was performed using the shade-dried fruit samples, with a final moisture content below 7%. Elemental concentrations were expressed on a dry-weight basis (mg kg^−1^ DW). Approximately 0.10 g of each dried homogenized sample was accurately weighed into a microwave digestion vessel, and 10 mL of concentrated nitric acid (69%, Carlo Erba Reagents S.r.l., Cornaredo, Italy) was added. After digestion, the clear solutions were quantitatively transferred into 50 mL volumetric flasks and diluted to volume with Milli-Q deionized water.

Quantification was performed by external calibration using a certified multi-element standard solution at six concentration levels (0.5, 1.0, 2.0, 6.0, 10.0, and 24.0 mg L^−1^). Calibration curves demonstrated excellent linearity, with correlation coefficients (r) ≥0.999 for all analyzed elements. The corresponding ICP-OES calibration curves, regression equations, and correlation coefficients are provided in Supplementary Figure S4. Calibration performance and instrumental stability were verified using independently prepared quality-control (QC) standard solutions at 8.0 and 16.0 mg L^−1^, which were not included among the calibration levels. The QC solutions were analyzed after calibration and periodically throughout the analytical sequence, and the measured concentrations remained within ±10% of their nominal values for all monitored elements.

Element-specific method limits of detection (LODs) were determined as three times the standard deviation (3σ) of ten replicate measurements of the digestion blank and were expressed on a dry-weight sample basis (mg kg^−1^ DW). The method LODs were: Ba, 0.008; Cd, 0.010; Cu, 0.012; Mn, 0.012; Zn, 0.015; Co, 0.015; Ni, 0.018; Pb, 0.020; Fe, 0.025; Ca, 0.030; Mg, 0.030; B, 0.040; As, 0.080; Se, 0.100, and K, 0.150 mg kg^−1^ DW. A matrix-matched certified reference material (CRM) was not available for inclusion in the present analytical sequence; therefore, analytical performance was additionally monitored using independently prepared QC standard solutions as described above.

The concentrations of As, B, Ba, Ca, Cd, Co, Cu, Fe, K, Mg, Mn, Ni, Pb, Se, and Zn were determined. Emission intensities were monitored at the following analytical wavelengths (nm): As (188.980), B (249.772), Ba (455.403), Ca (396.847), Cd (226.502), Co (238.892), Cu (327.395), Fe (238.204), K (766.491), Mg (279.553), Mn (257.610), Ni (230.299), Pb (220.353), Se (196.026), and Zn (213.857). Elemental concentrations were expressed as mg kg^−1^ dry weight (DW). Each sample was digested once, and the resulting digested sample solution was measured three times by ICP-OES. These measurements were technical (instrumental) replicates of the same digest and do not represent independent biological replicates. The mean instrumental response was used for quantification.

### 2.6. Determination of DPPH Radical-Scavenging Activity

The antiradical activity of fruit extracts was evaluated using the DPPH (2,2-diphenyl-1-picrylhydrazyl) radical scavenging assay according to the method described by Cosmulescu et al. [29,30], with minor modifications.

For extract preparation, 1.0 g of each fruit sample was extracted with 10 mL of 70% ethanol for 60 min in the dark with periodic mixing. The extracts were subsequently filtered and stored at 4 °C in the dark until analysis. The filtered extracts were used directly in the DPPH assay without further dilution. A freshly prepared 0.1 mM DPPH solution in methanol was equilibrated in the dark for 1 h before use. The reaction mixture consisted of 1 mL of the undiluted fruit extract and 3 mL of DPPH solution. A control containing 1 mL of solvent and 3 mL of DPPH solution was prepared in parallel. Because of the natural pigmentation of the fruit extracts, a sample blank containing 1 mL of extract and 3 mL of 70% ethanol was used to correct for background absorbance. After incubation for 30 min in the dark at room temperature, absorbance was measured at 517 nm using a UV-1800PC UV-Vis spectrophotometer (SCILOGEX, Rocky Hill, CT, USA).

DPPH radical scavenging activity was calculated according to the following equation:

DPPH inhibition (%) = *A*_control_ − (*A*_sample_ − *A*_blank_)/*A*_control_ × 100

where *A*_control_ is the absorbance of the DPPH control, *A*_sample_ is the absorbance of the extract-DPPH reaction mixture, and *A*_blank_ is the background absorbance of the corresponding sample. L-ascorbic acid (0.0332 mg mL^−1^) was used as a reference antioxidant.

For rosehip, DPPH analysis was performed on two selected paired samples, RC-4 (Sevan) and RC-6 (Goght), for which both fresh and corresponding shade-dried materials were available. These two samples were selected because they showed the highest vitamin C concentrations among the three shade-dried rosehip samples analyzed (RC-4, RC-6, and RC-7). The selection was targeted rather than random and was intended to provide an exploratory comparison of DPPH radical-scavenging activity between the corresponding fresh and shade-dried materials. All four fresh sea buckthorn samples were analyzed under the same experimental conditions.

Replicate measurements in the HPLC, DPPH, and ICP-OES analyses were technical replicates obtained from the same prepared sample extract or digested solution and did not represent independent biological replicates. Accordingly, these replicate measurements reflect analytical measurement repeatability rather than biological or population-level variability. However, the individual technical replicate readings for the ICP-OES and DPPH analyses are no longer available. Consequently, the analytical variability of these measurements, including standard deviations (SD) and relative standard deviations (RSD), cannot be calculated retrospectively or reported. This limitation should be considered when interpreting the corresponding results. Data processing and descriptive analysis were performed using Microsoft Excel. No inferential statistical tests were applied because independent biological replicates were not available for the individual sample codes. Calibration curves for the chromatographic and ICP-OES analyses were constructed by linear regression. For the chromatographic analyses, coefficients of determination (R^2^) were used to evaluate linearity, whereas correlation coefficients (r) were used for the ICP-OES calibration curves.

## 3. Results

### 3.1. Soluble Sugar and Vitamin C Profiles of Rosehip and Sea Buckthorn Fruits

**Figure 1:**
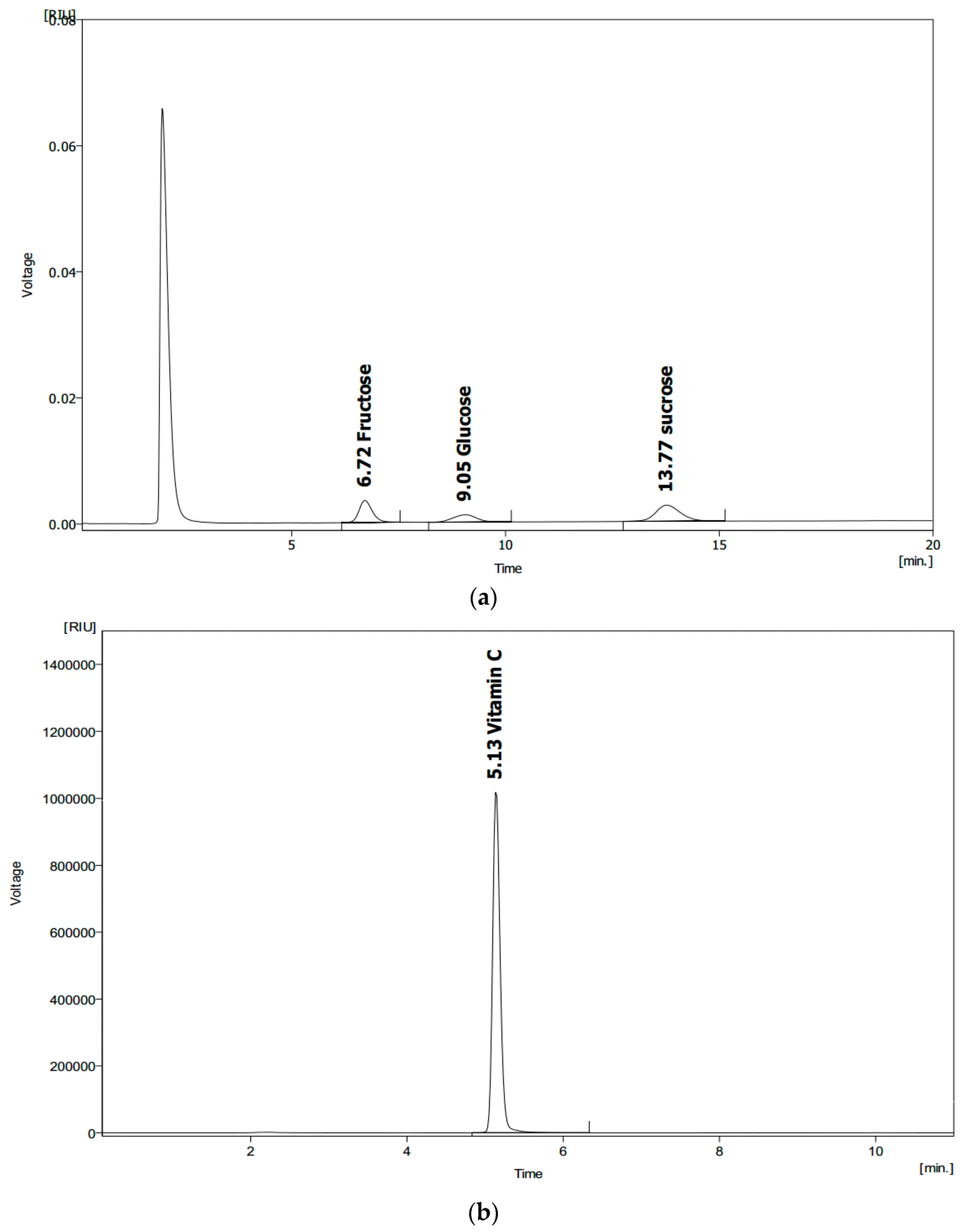
Representative HPLC chromatograms of reference standards showing the retention times of the target analytes under the applied chromatographic conditions: (**a**) HPLC-RID chromatogram of the fructose, glucose, and sucrose standard mixture; (**b**) HPLC-DAD chromatogram of the L-ascorbic acid standard.

The HPLC methods provided adequate separation and quantification of the target compounds. The chromatographic performance of the developed methods was first evaluated using reference standards. The HPLC-RID chromatogram of the standard sugar mixture showed well-resolved peaks for fructose, glucose, and sucrose, while the HPLC-DAD analysis of the ascorbic acid standard produced a distinct chromatographic peak under the selected conditions (Figure 1).

Good linearity was obtained for fructose, glucose, and sucrose (R^2^ = 0.9998, 0.9970, and 0.9994, respectively), while the ascorbic acid calibration showed R^2^ > 0.9999. Detailed calibration data and calibration curves are provided in the Supplementary Materials Table S1 and Figure S2, respectively, while representative HPLC chromatograms are shown in Figure S3.

The soluble sugar and vitamin C profiles of the investigated fruit samples are summarized in Table 2.

The measured soluble sugar concentrations differed among the fresh rosehip samples. Fructose ranged from 5.98 to 18.19 g/100 g, while glucose ranged from 4.81 to 18.48 g/100 g. The highest combined concentration of the two detected sugars was observed in RC-1 (Hankavan; 36.67 g/100 g), whereas RC-3 (Sevqar) showed the lowest value (10.79 g/100 g). Fructose predominated over glucose in all analyzed fresh rosehip samples except RC-1, in which glucose (18.48 g/100 g) slightly exceeded fructose (18.19 g/100 g).

Among the shade-dried rosehip samples, RC-4 (Sevan) showed the highest concentrations of fructose and glucose, at 26.27 and 18.35 g/100 g, respectively, corresponding to a combined concentration of 44.62 g/100 g. This was followed by RC-6 (Goght), with 38.20 g/100 g, and RC-7 (Aparan), with 28.36 g/100 g. Sucrose was not detected in any of the analyzed fresh or shade-dried rosehip samples.

The measured vitamin C concentrations ranged from 130 to 900 mg/100 g among the fresh rosehip samples. The highest concentration was found in RC-6 (Goght; 900 mg/100 g), followed by RC-2 (Geghashen; 680 mg/100 g), RC-4 (Sevan; 480 mg/100 g), RC-1 (Hankavan; 370 mg/100 g), RC-7 (Aparan; 272 mg/100 g), and RC-5 (Drakhtik; 200 mg/100 g). The lowest concentration was recorded in RC-3 (Sevqar; 130 mg/100 g). Among the shade-dried samples, vitamin C concentrations were 670, 650, and 374 mg/100 g in RC-6 (Goght), RC-4 (Sevan), and RC-7 (Aparan), respectively. Overall, the analyzed rosehip samples showed a broad range of measured soluble sugar and vitamin C concentrations.

The four fresh sea buckthorn samples exhibited a consistent soluble sugar pattern, with fructose predominating over glucose in all samples. Fructose concentrations ranged from 0.251 to 0.523 g/100 g, while glucose ranged from 0.055 to 0.208 g/100 g. SB-3 showed the highest concentrations of both fructose (0.523 g/100 g) and glucose (0.208 g/100 g), corresponding to a combined concentration of 0.731 g/100 g. Sucrose was not detected in any of the analyzed sea buckthorn samples.

Vitamin C concentrations in fresh sea buckthorn fruits ranged from 11.9 to 40.0 mg/100 g. The highest concentration was detected in SB-2 (40.0 mg/100 g), followed by SB-4 (21.0 mg/100 g) and SB-1 (17.0 mg/100 g), whereas SB-3 exhibited the lowest concentration (11.9 mg/100 g). Overall, the measured soluble sugar and vitamin C concentrations differed among the four analyzed sea buckthorn samples.

### 3.2. Elemental Profiles of Rosehip and Sea Buckthorn Fruits

ICP-OES analysis showed differences in the measured elemental concentrations among the investigated rosehip and sea buckthorn samples (Table 3). Among the quantified elements, K, Ca, and Mg were the predominant mineral constituents. The values reported for each sample represent the mean of three technical instrumental readings of a single digested sample solution and therefore do not represent biological variability.

All quantified concentrations of As, Cd, and Pb were above their respective method LODs (0.080, 0.010, and 0.020 mg kg^−1^ DW, respectively).

The measured Ca and Mg concentrations were numerically higher in the analyzed rosehip samples than in the analyzed sea buckthorn samples. In rosehip, K ranged from 7424.53 to 16,880.78 mg kg^−1^ DW, with the highest concentration observed in RC-3 (Sevqar). Calcium concentrations ranged from 4954.27 to 10,826.43 mg kg^−1^ DW, with the maximum recorded in RC-7 (Aparan), while Mg varied from 1411.70 to 3474.68 mg kg^−1^ DW. The measured Mn concentrations also differed among the rosehip samples, with the highest value observed in RC-3 (105.55 mg kg^−1^ DW). The measured K, Ca, Mg, and Mn concentrations also differed among the investigated rosehip samples.

Sea buckthorn displayed a different elemental pattern. Potassium remained the predominant element, ranging from 8125.75 to 10,662.91 mg kg^−1^ DW, while the measured Ca and Mg concentrations were numerically lower than those in the analyzed rosehip samples. Higher measured Fe concentrations were observed in the analyzed sea buckthorn samples than in the analyzed rosehip samples, with the highest value recorded in SB-2 (86.08 mg kg^−1^ DW). Among the sea buckthorn samples, SB-4 had the highest measured Cu and Zn concentrations, at 7.07 and 26.76 mg kg^−1^ DW, respectively.

With respect to potentially toxic elements, Pb was not detected in any rosehip sample, whereas Cd was detected in several rosehip samples at concentrations up to 2.09 mg kg^−1^ DW. Arsenic concentrations ranged from non-detectable levels to 7.85 mg kg^−1^ DW among the rosehip samples. In sea buckthorn, Cd and Ni were not detected, while Pb was detected only in selected samples. Overall, the measured elemental concentrations differed between the analyzed rosehip and sea buckthorn samples and among individual samples within each fruit group.

### 3.3. DPPH Radical-Scavenging Activity of Rosehip and Sea Buckthorn Fruits

The DPPH radical-scavenging activity of the investigated rosehip and sea buckthorn fruit samples is presented in Table 4. In the two selected rosehip sample pairs, the shade-dried materials showed lower measured DPPH radical-scavenging activity than their corresponding fresh materials, whereas the four analyzed fresh sea buckthorn samples showed DPPH inhibition values within a narrow range.

Among the two selected fresh rosehip samples, DPPH inhibition was 86.22% for RC-4 (Sevan) and 67.54% for RC-6 (Goght). The corresponding shade-dried samples showed inhibition values of 3.37% and 9.80%, respectively.

The four fresh sea buckthorn samples showed DPPH inhibition values within a narrow range of 94.90–95.18%. The highest measured inhibition was observed for SB-1 (95.18%), followed by SB-4 (95.13%), SB-3 (94.95%), and SB-2 (94.90%).

Overall, lower DPPH inhibition values were measured in the shade-dried materials of the two selected rosehip sample pairs than in their corresponding fresh materials, whereas the four fresh sea buckthorn samples showed similar measured inhibition values under the applied assay conditions. These comparisons are descriptive because independent biological replicates were not available. The measured DPPH radical-scavenging activity did not consistently parallel the vitamin C concentrations reported in Table 2, indicating that vitamin C content alone did not account for the observed DPPH response.

## 4. Discussion

The present study provides a descriptive comparison of the phytochemical composition, elemental profiles, and DPPH radical-scavenging activity of rosehip and sea buckthorn fruits collected from different locations in Armenia. However, given the differences in botanical identity, sampling structure, and collection periods, these observed differences cannot be attributed solely to geographical origin and should be interpreted at the sample level. Distinct compositional patterns were observed between the two fruit groups, while considerable variation was also evident among samples collected from different localities. In the case of rosehip, taxonomic variation should additionally be considered, since most samples were identified as *Rosa canina* L., whereas the Geghashen and Sevqar samples were identified as *Rosa corymbifera* Borkh. and *Rosa nisami* Sosn., respectively. Previous studies comparing different *Rosa* species have demonstrated substantial interspecific variability in ascorbic acid, phenolic compounds, carotenoids, mineral composition, and antioxidant activity [4,6,11]. Thus, the differences observed in the present study most likely reflect the combined influence of geographical origin, environmental conditions, taxonomic background, fruit maturity, and other biological factors rather than a single determinant.

### 4.1. Soluble Sugars and Vitamin C

Fructose and glucose were the predominant soluble sugars detected in the investigated fruits, whereas sucrose was not detected under the applied analytical conditions. In the analyzed samples, the measured concentrations of both monosaccharides were numerically higher in rosehip than in sea buckthorn. Among the fresh rosehip samples, RC-1 (Hankavan) exhibited the highest combined concentration of fructose and glucose, whereas RC-3 (Sevqar) showed the lowest concentrations. The measured soluble carbohydrate concentrations differed among the investigated rosehip samples.

The measured fructose and glucose concentrations, expressed per 100 g of sample, were numerically higher in the shade-dried rosehip samples than in their corresponding fresh materials. This difference should primarily be interpreted as a concentration effect associated with water removal during drying rather than as evidence of an absolute increase in sugar content. Consequently, direct comparison between fresh- and dry-weight samples requires caution because their moisture contents differ substantially.

The measured vitamin C concentrations ranged from 130 to 900 mg/100 g among the investigated fresh rosehip samples. The highest concentration was recorded in RC-6 (Goght), followed by RC-2 (Geghashen), whereas RC-3 (Sevqar) contained the lowest amount. Wide variation in ascorbic acid content among rosehip fruits has also been reported previously. Ercisli reported vitamin C concentrations of 727–943 mg/100 g fresh weight (FW) among different *Rosa* species [4], while Medveckienė et al. demonstrated that vitamin C accumulation can vary considerably according to species/cultivar and fruit ripening stage [9]. The concentration measured in the Goght sample (900 mg/100 g) therefore falls within the range reported for rosehip in previous studies, while the other investigated samples showed a broad range of measured vitamin C concentrations.

Taxonomic identity may be one of several factors contributing to the observed differences among the samples. Fascella et al. reported significant differences in ascorbic acid, phenolic compounds, carotenoids, and antioxidant activity among fruits of *R. canina*, *R. corymbifera*, *R. micrantha*, and *R. sempervirens* [6]. Accordingly, the particularly low vitamin C concentration detected in RC-3 should not be interpreted solely as a geographical effect, since this sample was botanically identified as *R. nisami*, whereas RC-2 belonged to *R. corymbifera*. Differences in measured vitamin C concentrations were also observed among samples identified as *R. canina*; therefore, the present descriptive dataset does not allow the contribution of taxonomic identity to be separated from other sample-specific factors.

In the analyzed sea buckthorn samples, the measured vitamin C concentrations were numerically lower than in the analyzed rosehip samples, ranging from approximately 11.9 to 40.0 mg/100 g. The chemical composition of *Hippophae rhamnoides* fruits is known to be highly variable, and broad differences in vitamin C and other bioactive compounds have been reported among cultivars, subspecies, geographical regions, developmental stages, and growing conditions [15]. The measured vitamin C concentrations in the investigated sea buckthorn samples may reflect multiple botanical, developmental, harvesting, and environmental factors. However, because these factors were not experimentally separated in the present study, their individual contributions cannot be determined.

### 4.2. Elemental Composition

Elemental profiling showed differences in the measured elemental concentrations between the analyzed rosehip and sea buckthorn samples and among individual samples within each fruit group. Potassium was one of the predominant elements in both fruit groups, while the measured Ca and Mg concentrations were numerically higher in the analyzed rosehip samples than in the analyzed sea buckthorn samples. The measured concentrations of K, Ca, Mg, Mn, and Ba also differed among the investigated rosehip samples. RC-3 (Sevqar), for example, showed the highest K and Mn concentrations, whereas RC-7 (Aparan) contained the highest Ca concentration.

The predominance of K, Ca, and Mg in rosehip is consistent with previous studies. Ercisli identified K, Ca, and Mg among the principal mineral constituents of *Rosa* fruits [4]. More recently, Milenković et al. investigated 52 fruit samples representing six wild *Rosa* species, including *R. canina* and *R. corymbifera*, and reported K concentrations of 4710–9850 mg/kg, Ca of 2650–5140 mg/kg, and Mg of 402–1000 mg/kg [12]. Several Armenian rosehip samples in the present study showed measured Ca and Mg concentrations extending above the ranges reported in that study.

The K concentrations observed in the present rosehip samples are also comparable in magnitude to those reported by Medveckienė et al., who found K to be the predominant macroelement in several rosehip species and cultivars [13]. Importantly, that study demonstrated that both species/cultivar and ripening stage influenced mineral concentrations [11]. These findings reinforce the need for caution when interpreting differences among geographically separated plant samples, as elemental composition may reflect multiple interacting biological and environmental factors.

Sea buckthorn exhibited a distinctly different elemental pattern. Although K was again the predominant element, the measured Ca and Mg concentrations were numerically lower in the analyzed sea buckthorn samples than in the analyzed rosehip samples. Conversely, higher measured Fe concentrations were observed in the analyzed sea buckthorn samples than in the analyzed rosehip samples, with the highest value recorded in SB-2. Differences among the four sea buckthorn samples were also apparent for Fe, Zn, Cu, and several other trace elements. Sea buckthorn fruits have previously been described as complex matrices containing a broad range of essential mineral elements, with composition varying according to geographical origin, cultivar, maturity, and environmental conditions [17].

The elemental differences observed among the investigated samples may reflect the combined influence of multiple biological and environmental factors. Mineral uptake and accumulation in plants are affected by variables including soil mineral composition, element bioavailability, soil pH, climatic conditions, water availability, plant genotype, and physiological stage. Since soil composition and other environmental variables were not independently measured in the present study, and the sampling design included differences in botanical identity and collection structure, the observed elemental differences cannot be attributed specifically to geographical origin or to any individual environmental parameter.

From the perspective of xenobiotic relevance, particular attention should be given to the non-essential elements detected in the investigated fruits. As, Ba, Cd, and Pb showed different patterns of occurrence and measured concentrations among the investigated samples. Pb was not detected in the investigated rosehip samples, whereas Cd and As were detected at different measured concentrations among individual samples. In sea buckthorn, Cd was not detected, while Pb was detected only in selected samples. Among the rosehip samples, the measured Ba concentrations ranged from 2.86 to 56.95 mg/kg DW, with the highest value recorded in RC-7. The occurrence and variability of these non-essential elements provide a direct xenobiotic-relevant dimension to the elemental characterization of the investigated medicinal and edible plant materials.

Among the potentially toxic elements, As, Cd, and Pb showed sample-dependent occurrence among the investigated materials. As and Cd were quantified in several rosehip samples, whereas Pb was not detected in the analyzed rosehip samples. In sea buckthorn, As was quantified in SB-2 and SB-3 and Pb in SB-1 and SB-4, whereas Cd was not detected. These findings are interpreted descriptively at the individual-sample level. Their toxicological significance cannot be inferred from concentration data alone, because exposure levels, consumption amounts, bioavailability, and applicable regulatory limits were not evaluated in the present study.

### 4.3. DPPH Radical-Scavenging Activity

The DPPH radical-scavenging results showed different measured responses among the investigated fruit materials. The two selected fresh rosehip samples showed DPPH inhibition values of 86.22% for RC-4 and 67.54% for RC-6. In both selected rosehip sample pairs, lower DPPH inhibition values were measured in the shade-dried materials than in their corresponding fresh materials. For RC-4 (Sevan), DPPH inhibition was 86.22% in the fresh material and 3.37% in the corresponding shade-dried material, while the respective values for RC-6 (Goght) were 67.54% and 9.80%. Because these comparisons were based on two selected sample pairs without independent biological replication, they should be interpreted descriptively and do not establish a statistically supported effect of drying.

The observed DPPH responses did not consistently parallel the measured vitamin C concentrations. Fresh RC-6 contained 900 mg/100 g vitamin C but exhibited lower DPPH inhibition than fresh RC-4, which contained 480 mg/100 g. Similarly, the shade-dried RC-4 and RC-6 samples contained relatively similar vitamin C concentrations (650 and 670 mg/100 g, respectively), while their DPPH inhibition values differed considerably. These observations indicate that vitamin C concentration alone did not account for the measured DPPH responses of the investigated rosehip extracts.

This interpretation is supported by the complex phytochemical composition of rosehip. Tumbas et al. demonstrated that *R. canina* contains several groups of antioxidant constituents, including vitamin C, flavonoids, and phenolic acids, which contribute to its free-radical-scavenging properties [14]. Other investigations have similarly demonstrated that rosehip fruits contain diverse phenolic compounds with substantial antioxidant potential [15]. Moreover, differences in antioxidant activity among *Rosa* species have been associated with variation in phenolic compounds, carotenoids, anthocyanins, and ascorbic acid [6]. Consequently, antioxidant constituents other than ascorbic acid may also have contributed to the measured DPPH responses; however, their individual contributions were not determined in the present study.

The lower DPPH inhibition values measured in the two shade-dried rosehip samples may be related to changes in antioxidant constituents and/or their extractability during processing; however, the present data do not allow the specific cause of these differences to be established. Phenolic compounds, flavonoids, carotenoids, and other potentially contributing antioxidant constituents were not quantitatively determined in the present study. Further targeted phytochemical analyses and appropriately replicated drying experiments would be required to investigate the mechanisms underlying the observed differences between the selected fresh and shade-dried samples.

A different pattern was observed for sea buckthorn. All four fresh sea buckthorn samples showed DPPH inhibition values within a narrow range of 94.90–95.18%, despite differences in their measured vitamin C concentrations. Sea buckthorn fruits are reported to contain multiple classes of antioxidant compounds, including phenolics, flavonoids, carotenoids, tocopherols, and ascorbic acid [22]. Such constituents may contribute to DPPH radical-scavenging activity; however, their contribution was not quantified in the present samples.

The very narrow range of DPPH inhibition among the sea buckthorn samples should nevertheless be interpreted cautiously. All samples produced approximately 95% inhibition under the selected experimental conditions, indicating that the assay was operating in a high-inhibition region. DPPH measurements are influenced by factors including antioxidant concentration, the DPPH-to-antioxidant ratio, reaction conditions, and the presence of pigments, which may limit discrimination among highly active extracts [31]. Therefore, the present results indicate high measured DPPH inhibition under the applied assay conditions but do not establish equivalent overall antioxidant capacities among the four sea buckthorn samples.

### 4.4. Overall Interpretation and Study Limitations

Taken together, the results provide a descriptive comparison of the chemical profiles of the investigated Armenian rosehip and sea buckthorn fruits and show differences in the measured parameters among individual samples. Within the analyzed sample set, the rosehip samples showed numerically higher measured soluble sugar, vitamin C, Ca, and Mg concentrations than the analyzed sea buckthorn samples. The analyzed sea buckthorn samples showed lower measured soluble sugar and vitamin C concentrations than the analyzed rosehip samples, while their DPPH inhibition values ranged from 94.90% to 95.18% under the applied assay conditions.

No single measured parameter adequately described the overall chemical and DPPH radical-scavenging characteristics of the investigated samples. Vitamin C concentration did not consistently parallel the measured DPPH radical-scavenging activity, while the elemental concentrations differed among the investigated samples. These observations support an integrated analytical approach to the characterization of medicinal plant-derived materials rather than evaluation based on a single chemical marker.

Several limitations should nevertheless be considered. First, the investigated rosehip material included three *Rosa* taxa; therefore, some of the observed differences among samples may be related to taxonomic identity rather than geographical origin. Second, environmental parameters such as soil composition, soil pH, irrigation or water characteristics, altitude-related climatic variables, and microclimatic conditions were not measured simultaneously; consequently, causal relationships between specific environmental factors and fruit composition cannot be established. Third, DPPH analysis was performed on selected rosehip samples rather than the complete rosehip collection, and independent biological replicates were not available for the individual sample codes. Therefore, the observed fresh-versus-shade-dried differences should be regarded as descriptive and exploratory rather than as evidence of a statistically supported effect of drying. Finally, additional antioxidant constituents, particularly phenolics, flavonoids, and carotenoids, were not quantified; therefore, their contribution to the measured DPPH responses and to the differences between fresh and shade-dried materials cannot be established from the present data.

Despite these limitations, the present study provides a comparative chemical dataset for rosehip and sea buckthorn fruits collected from different locations in Armenia. The observed differences in vitamin C, soluble sugars, elemental composition, and DPPH radical-scavenging activity highlight the importance of considering botanical identity and sample processing when characterizing medicinal plant-derived materials. Future investigations incorporating balanced sampling designs, independent biological replicates, broader phytochemical profiling, and simultaneous characterization of soil, climatic, and other environmental parameters would help distinguish the relative contributions of botanical and environmental factors to the observed compositional differences.

## 5. Conclusions

This study provides an integrated characterization of the phytochemical composition, essential and non-essential elemental profiles, and DPPH radical-scavenging activity of rosehip (*Rosa* spp.) and sea buckthorn (*Hippophae rhamnoides* L.) fruits collected from different locations in Armenia. Differences in the measured compositional parameters were observed between the analyzed fruit groups and among individual samples. Within the analyzed sample set, the measured soluble sugar, vitamin C, Ca, and Mg concentrations were numerically higher in rosehip than in sea buckthorn, whereas the four analyzed fresh sea buckthorn samples showed DPPH inhibition values within a narrow range of 94.90–95.18% under the applied assay conditions.

Particular attention was given to potentially toxic elements because of their relevance to elemental exposure. As, Cd, and Pb showed sample-dependent occurrence among the investigated materials. These concentrations are reported descriptively and should not be interpreted as direct indicators of toxicological risk or product safety, because exposure levels, consumption amounts, bioavailability, and applicable regulatory thresholds were not evaluated.

The measured DPPH radical-scavenging activity did not consistently parallel vitamin C content, indicating that vitamin C alone did not account for the observed DPPH responses. Other antioxidant constituents may also contribute; however, their individual contributions were not determined in the present study. In the two selected rosehip sample pairs, lower DPPH inhibition values were measured in the shade-dried materials than in the corresponding fresh materials. However, because the comparison was based on selected samples without independent biological replication, these observations are descriptive and do not establish a statistically supported effect of drying.

Overall, the findings demonstrate substantial chemical heterogeneity among the investigated Armenian rosehip and sea buckthorn fruits and highlight the importance of botanical identity, sample processing, and elemental profiling in their characterization. Given the differences in botanical identity, sampling structure, and collection periods, the observed compositional differences should be interpreted at the sample level and cannot be attributed specifically to geographical origin. Because environmental exposure pathways were not directly assessed, the sources of the observed non-essential elements cannot be established from the present dataset. Future studies incorporating balanced sampling designs, independent biological replicates, broader phytochemical characterization, and simultaneous assessment of soil, water, climatic, and other environmental parameters would help clarify the relative contributions of botanical and environmental factors to the observed compositional differences and the potential sources of non-essential element exposure.

## Figures and Tables

**Table 1:** Sampling sites and botanical identification of the investigated rosehip and sea buckthorn fruit samples collected in Armenia.

Sample Code	Plant Material	Botanical Identification	Province	Locality	Latitude (N)	Longitude (E)	Collection Year
RC-1	Rosehip	*Rosa canina* L.	Kotayk	Hankavan	40°37′15.5″	44°38′28.2″	2025
RC-2		*Rosa corymbifera* Borkh.	Kotayk	Geghashen	40°17′45.2″	44°41′32.6″	2025
RC-3		*Rosa nisami* Sosn.	Tavush	Sevqar	41°01′18.2″	45°14′37.0″	2025
RC-4		*Rosa canina* L.	Gegharkunik	Sevan	40°32′24.4″	44°57′58.8″	2025
RC-5		*Rosa canina* L.	Gegharkunik	Drakhtik	40°32′38.7″	45°12′43.2″	2025
RC-6		*Rosa canina* L.	Kotayk	Goght	40°14′52.0″	44°45′26.5″	2025
RC-7		*Rosa canina* L.	Aragatsotn	Aparan	40°35′22.0″	44°22′03.3″	2025
SB-1	Sea buckthorn	*H. rhamnoides* L.	Gegharkunik	Drakhtik	40°32′38.7″	45°12′43.2″	2024
SB-2		*H. rhamnoides* L.	Gegharkunik	Drakhtik	40°32′39.5″	45°12′44.6″	2024
SB-3		*H. rhamnoides* L.	Gegharkunik	Drakhtik	40°32′52.7″	45°12′26.5″	2024
SB-4		*H. rhamnoides* L.	Gegharkunik	Sevan Basin	40°32′42.9″	45°12′42.4″	2024

**Table 2:** Soluble sugar and vitamin C profiles of rosehip and sea buckthorn fruit samples collected from different locations in Armenia.

Sample Code	Sampling Locality	Sample State	Fructose (g/100 g)	Glucose (g/100 g)	Sucrose	Vitamin C (mg/100 g)
RC-1	Hankavan	Fresh	18.19 ± 0.55	18.48 ± 0.41	ND	370 ± 6
RC-2	Geghashen	Fresh	9.47 ± 0.18	7.40 ± 0.15	ND	680 ± 11
RC-3	Sevqar	Fresh	5.98 ± 0.12	4.81 ± 0.13	ND	130 ± 8
RC-4	Sevan	Fresh	12.30 ± 0.81	8.44 ± 0.23	ND	480 ± 19
RC-5	Drakhtik	Fresh	9.04 ± 0.19	7.02 ± 0.26	ND	200 ± 12
RC-6	Goght	Fresh	10.24 ± 0.24	6.92 ± 0.55	ND	900 ± 14
RC-7	Aparan	Fresh	11.87 ± 0.51	6.98 ± 0.32	ND	272 ± 7
RC-4	Sevan	Shade-dried	26.27 ± 0.47	18.35 ± 1.02	ND	650 ± 6
RC-6	Goght	Shade-dried	22.81 ± 0.65	15.39 ± 0.95	ND	670 ± 12
RC-7	Aparan	Shade-dried	16.29 ± 0.04	12.07 ± 0.27	ND	374 ± 8
SB-1	Drakhtik	Fresh	0.251 ± 0.01	0.063 ± 0.01	ND	17.0 ± 1.5
SB-2	Drakhtik	Fresh	0.416 ± 0.02	0.089 ± 0.06	ND	40.0 ± 3.6
SB-3	Drakhtik	Fresh	0.523 ± 0.01	0.208 ± 0.03	ND	11.9 ± 0.7
SB-4	Sevan Basin	Fresh	0.329 ± 0.01	0.055 ± 0.01	ND	21.0 ± 1.7

Note: Values are expressed as mean ± SD of three technical replicate measurements (*n* = 3). ND, not detected.

**Table 3:** Elemental composition of shade-dried rosehip and sea buckthorn fruits from different sampling localities in Armenia (mg kg^−1^ DW).

Element	RC-1	RC-2	RC-3	RC-4	RC-5	RC-6	RC-7	SB-1	SB-2	SB-3	SB-4
As	7.85	2.71	2.37	ND	2.43	6.10	ND	ND	1.03	0.24	ND
B	17.43	21.83	21.87	18.76	17.26	16.52	35.18	16.36	21.76	16.75	22.89
Ba	6.27	16.54	7.93	2.86	4.23	15.81	56.95	1.34	1.83	1.10	1.22
Ca	9632.46	5502.20	9013.86	6760.82	5492.68	4954.27	10,826.43	711.95	868.13	629.12	1217.44
Cd	2.09	1.34	1.93	1.33	1.74	1.69	ND	ND	ND	ND	ND
Co	ND	ND	ND	0.05	ND	ND	ND	ND	0.02	0.06	0.17
Cu	2.55	2.05	0.13	2.39	0.85	0.02	2.28	1.86	3.06	2.11	7.07
Fe	4.73	9.94	15.71	4.97	10.95	7.83	11.29	55.62	86.08	41.93	37.93
K	7424.53	8528.45	16,880.78	13,549.60	9789.21	11,929.54	10,506.16	8125.75	9295.13	8190.71	10,662.91
Mg	3474.68	1841.78	2241.17	2102.64	1663.45	1411.70	2843.95	670.16	871.06	706.45	532.08
Mn	26.27	38.44	105.55	3.61	31.54	21.92	36.24	8.34	9.78	5.48	7.07
Ni	4.29	1.29	ND	1.91	ND	ND	ND	ND	ND	ND	ND
Pb	ND	ND	ND	ND	ND	ND	ND	0.49	ND	ND	0.69
Se	ND	1.82	ND	ND	ND	ND	27.66	33.54	25.99	27.65	19.67
Zn	4.94	10.89	6.45	10.20	5.05	5.31	5.02	8.85	16.10	8.28	26.76

Note: Elemental concentrations are expressed as mg kg^−1^ dry weight (DW). The reported values represent the mean of three technical instrumental replicate measurements (*n* = 3) of a single digested sample solution. However, the individual replicate readings are no longer available; therefore, analytical variability (SD or RSD) cannot be calculated retrospectively or reported. These technical replicates do not represent independent biological replicates. ND, not detected.

**Table 4:** DPPH radical-scavenging activity values of rosehip and sea buckthorn fruit samples.

Sample Code	Sample State	DPPH Inhibition (%)
RC-4 (Sevan)	Fresh	86.22
RC-4 (Sevan)	Shade-dried	3.37
RC-6 (Goght)	Fresh	67.54
RC-6 (Goght)	Shade-dried	9.80
SB-1	Fresh	95.18
SB-2	Fresh	94.90
SB-3	Fresh	94.95
SB-4	Fresh	95.13
Ascorbic acid	Reference	97.07

Note: DPPH radical-scavenging activity is expressed as percentage inhibition. The reported values represent the mean of three technical replicate measurements (*n* = 3) performed on the same prepared sample extract. However, the individual replicate readings are no longer available; therefore, analytical variability (SD or RSD) cannot be calculated retrospectively or reported. These technical replicates do not represent independent biological replicates.

## Data Availability

The original contributions presented in this study are included in the article/Supplementary Materials. Further inquiries can be directed to the corresponding author.

## Supplementary Materials

The following supporting information can be downloaded at https://www.mdpi.com/article/10.3390/jox16060202/s1. Figure S1: Representative field photographs, fruit samples, and herbarium voucher specimens of the investigated rosehip (*Rosa* spp.) and sea buckthorn (*Hippophae rhamnoides* L.) plant materials. Herbarium voucher specimens were deposited in the Herbarium of Yerevan State University (ERCB); Table S1: Calibration levels used for HPLC quantification of soluble sugars and ascorbic acid; Figure S2: Calibration curves used for HPLC quantification: (a) fructose; (b) glucose; (c) sucrose; (d) ascorbic acid; Figure S3: Representative HPLC chromatograms of rosehip and sea buckthorn fruit extracts used for soluble sugar and vitamin C determination: (a) HPLC-RID chromatogram of the rosehip sample RC-5 (Drakhtik) for soluble sugar analysis; (b) HPLC-RID chromatogram of the sea buckthorn sample SB-3 for soluble sugar analysis; (c) HPLC-DAD chromatogram of the rosehip sample RC-2 (Geghashen) for vitamin C determination; (d) HPLC-DAD chromatogram of the sea buckthorn sample SB-3 for vitamin C determination; Figure S4: ICP-OES calibration curves for the determination of As, B, Ba, Ca, Cd, Co, Cu, Fe, K, Mg, Mn, Ni, Pb, Se, and Zn. The corresponding linear regression equations and correlation coefficients (r) are shown for each element.
