# Supplementary material for: Phytochemical Composition, Elemental Profiles, Antioxidant Activity, and Xenobiotic Relevance of Rosehip and Sea Buckthorn Fruits from Armenia
Source: J Xenobiot. 2026 Oct 21;16(6):202. doi: 10.3390/jox16060202 (PMC13705843; doi:10.3390/jox16060202)
Supplement: Supplementary file 1 [file jox-16-00202-s001.zip › jox-4595365-supplementary.pdf]

Supplementary Materials: Phytochemical Composition, Elemental Profiles, Antioxidant Activity, and Xenobiotic Relevance of Rosehip and Sea Buckthorn Fruits from Armenia

Lusine Sahakyan, Avetis Tsaturyan, Ela Minasyan, Lusine Hayrapetyan, Ani Chakhoyan, Valodya Mirzoyan, Narine Zakaryan, Rosanna Palumbo, Caterina Vicidomini, Giovanni N. Roviello and Ashot Saghyan

Figure S1. Representative field photographs, fruit samples, and herbarium voucher specimens of the investigated rosehip (*Rosa* spp.) and sea buckthorn (*Hippophae rhamnoides* L.) plant materials. Herbarium voucher specimens were deposited in the Herbarium of Yerevan State University (ERCB).

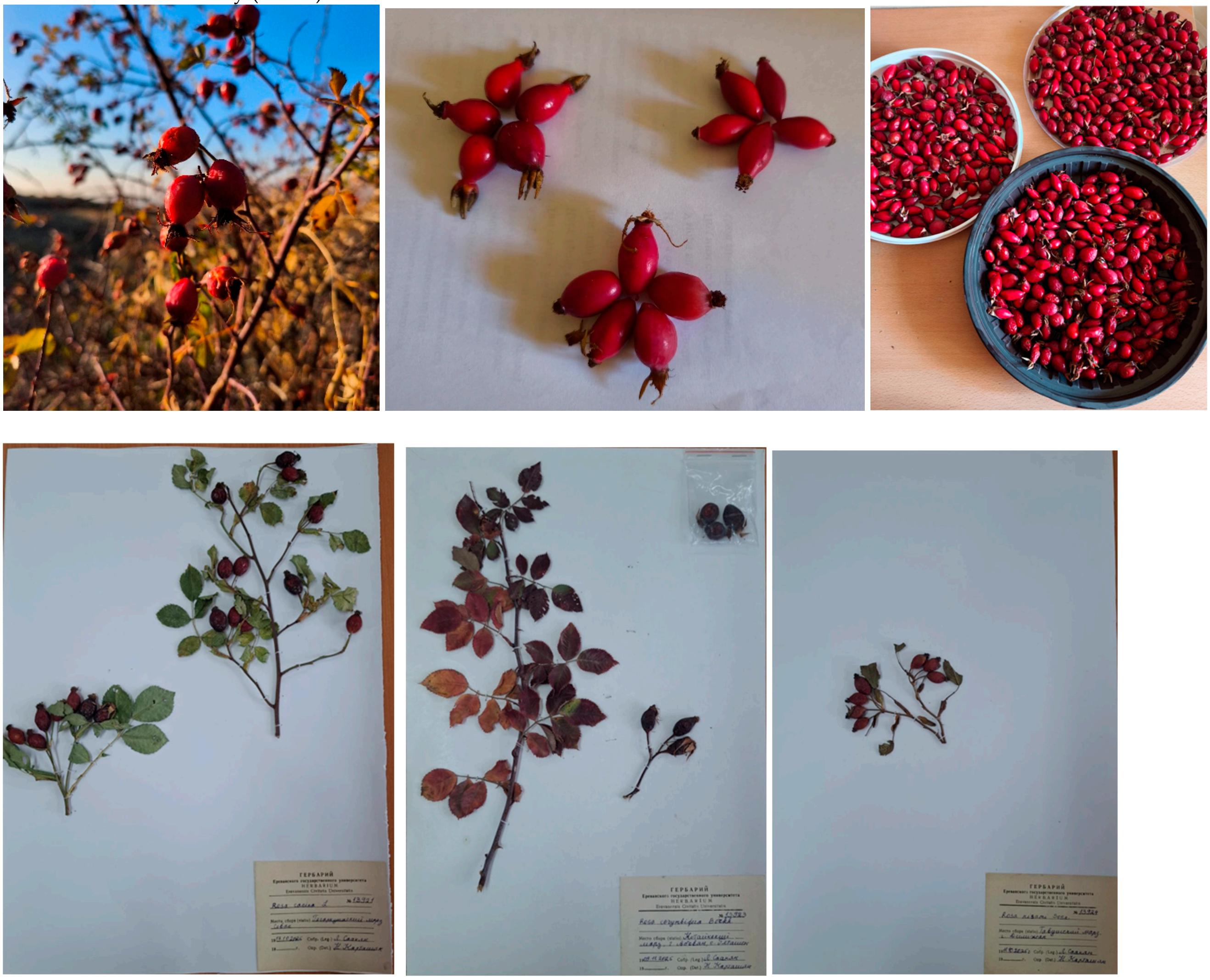

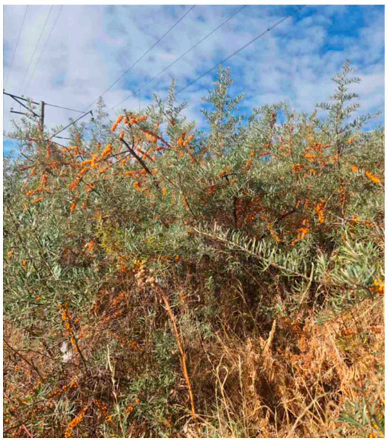
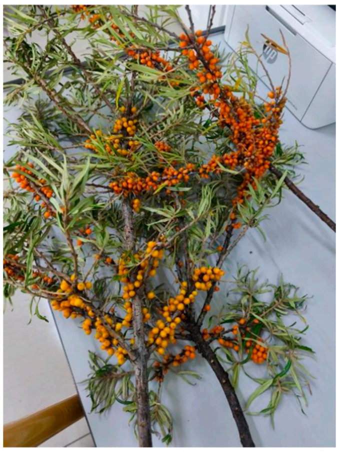
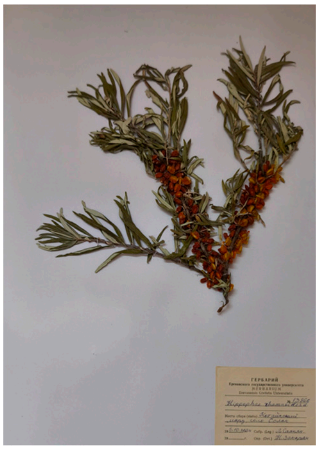
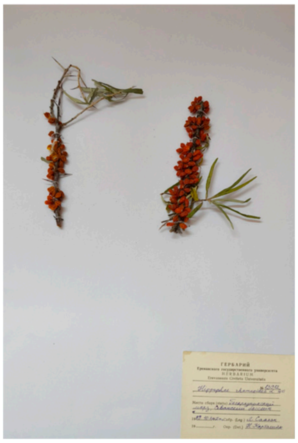

Table S1. Calibration levels used for HPLC quantification of soluble sugars and ascorbic acid

Calibration level	Fructose (mg/mL)	Glucose (mg/mL)	Sucrose (mg/mL)	Ascorbic acid (mg/mL)
Cal 1	2.077	2.683	2.450	0.2024
Cal 2	3.462	4.472	4.083	0.4048
Cal 3	6.923	8.943	8.167	0.8096
Cal 4	10.385	13.415	12.250	2.4288
Cal 5	20.770	26.830	24.500	7.2864

Figure S2. Calibration curves used for HPLC quantification:
(a) fructose; (b) glucose; (c) sucrose; (d) ascorbic acid.

(a)

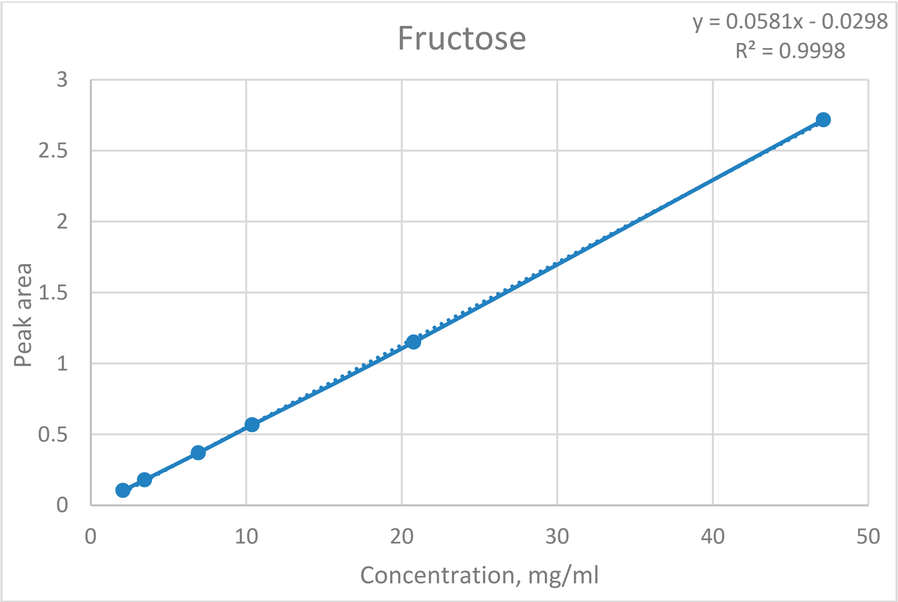

(b)

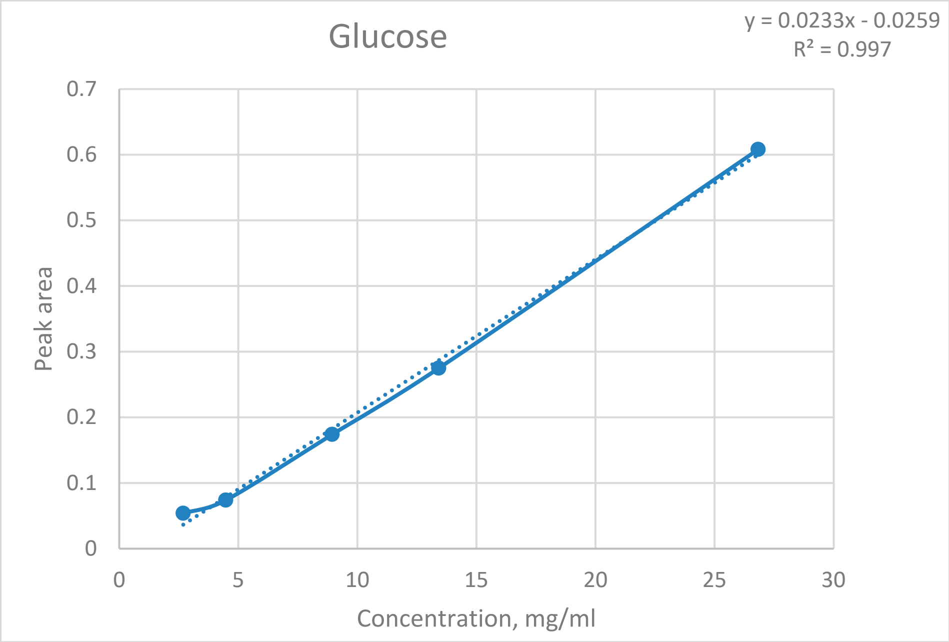
Glucose
y = 0.0233x - 0.0259
R² = 0.997
Peak area
0
0.1
0.2
0.3
0.4
0.5
0.6
0.7
0
5
10
15
20
25
30
Concentration, mg/ml

(c)

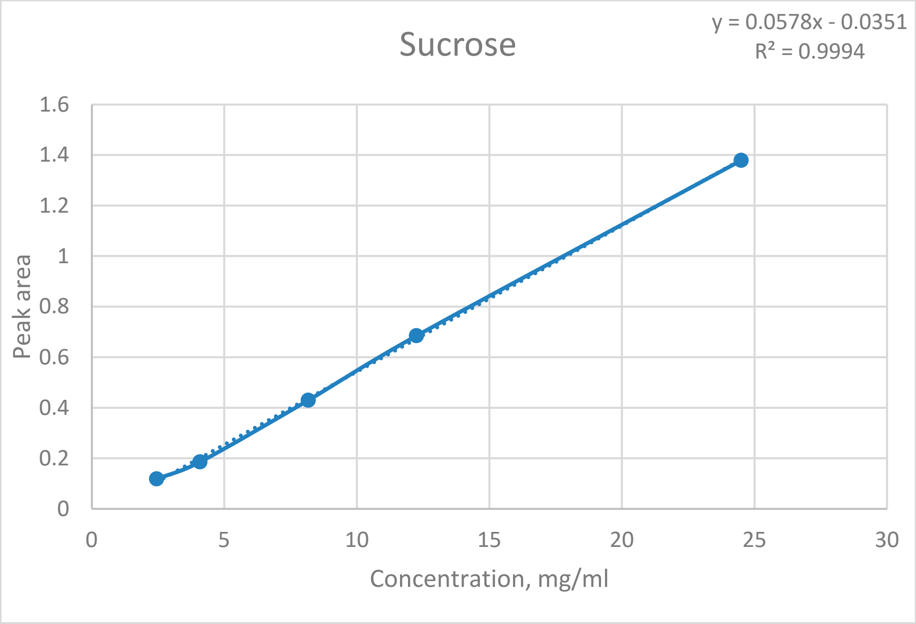
Sucrose
y = 0.0578x - 0.0351
R² = 0.9994
Peak area
0
0.2
0.4
0.6
0.8
1
1.2
1.4
1.6
0
5
10
15
20
25
30
Concentration, mg/ml

(d)

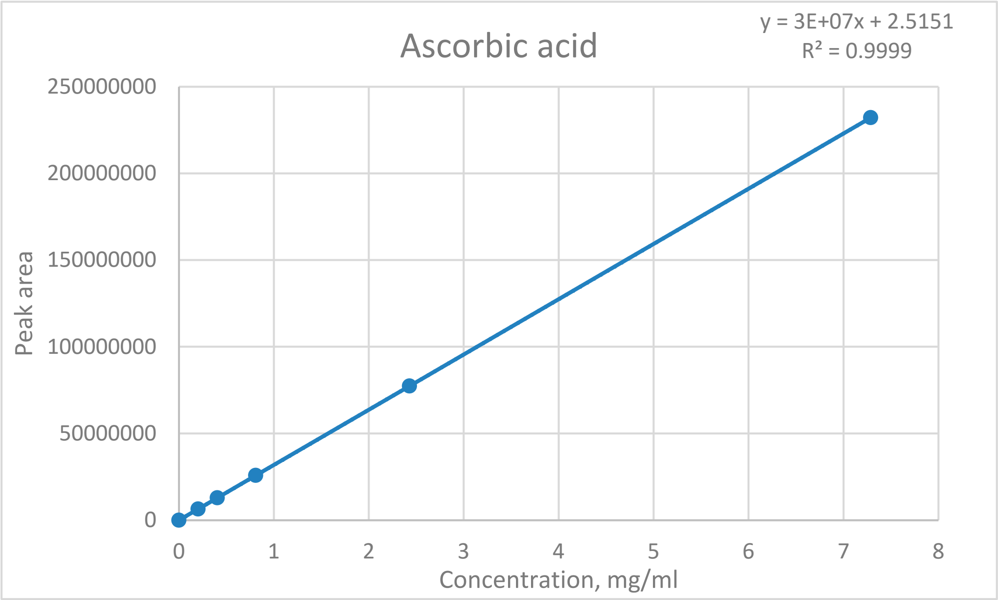

Figure S3. Representative HPLC chromatograms of rosehip and sea buckthorn fruit extracts used for soluble sugar and vitamin C determination: (a) HPLC-RID chromatogram of the rosehip sample RC-5 (Drakhtik) for soluble sugar analysis; (b) HPLC-RID chromatogram of the sea buckthorn sample SB-3 for soluble sugar analysis; (c) HPLC-DAD chromatogram of the rosehip sample RC-2 (Geghashen) for vitamin C determination; (d) HPLC-DAD chromatogram of the sea buckthorn sample SB-3 for vitamin C determination.

(a)

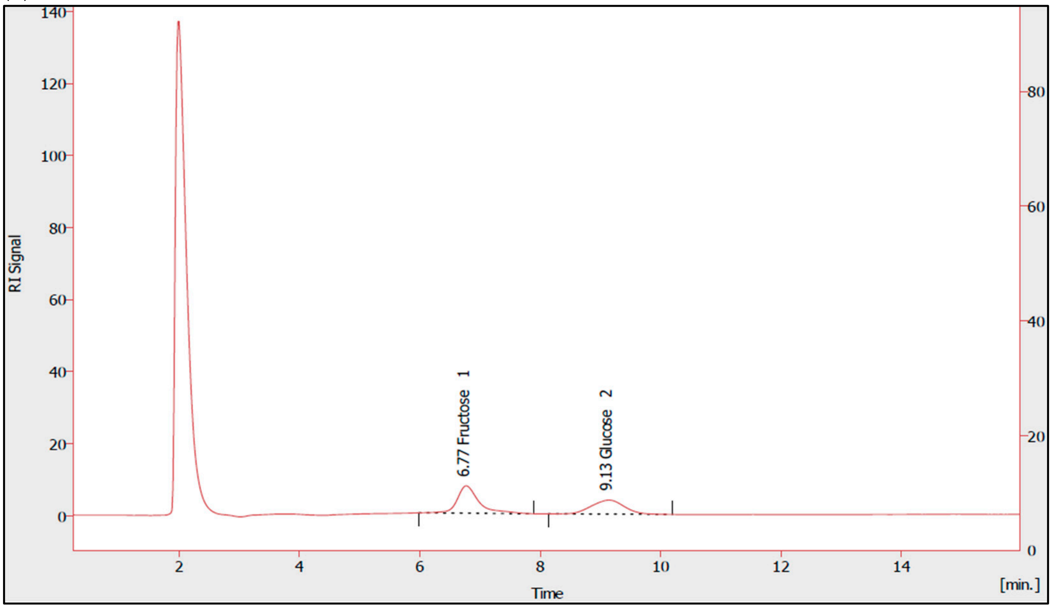

(b)

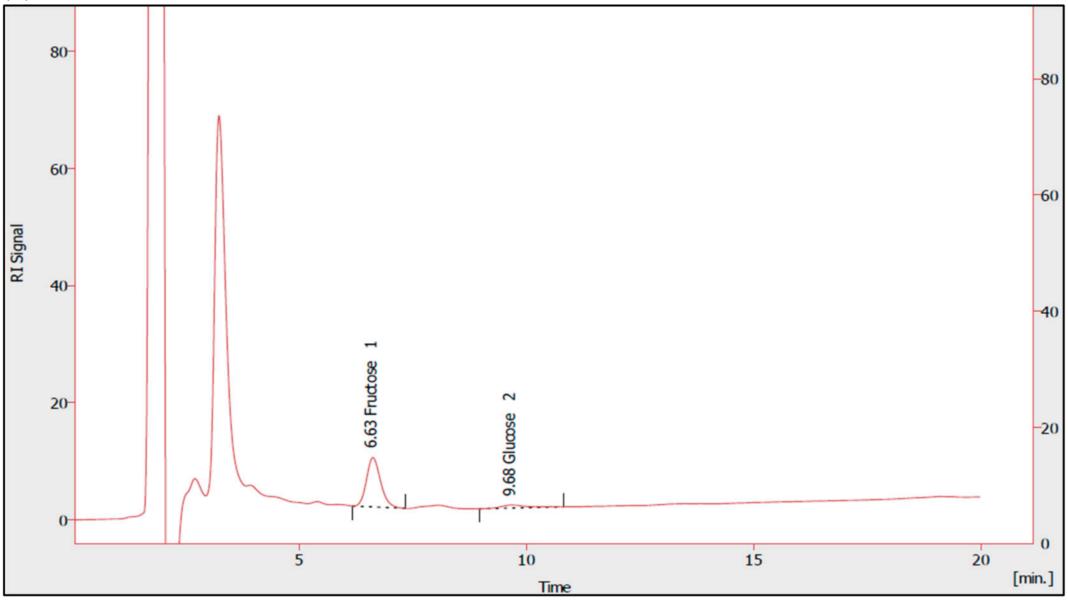
RI Signal
6.63 Fructose 1
9.68 Glucose 2
Time
[min.]

(c)

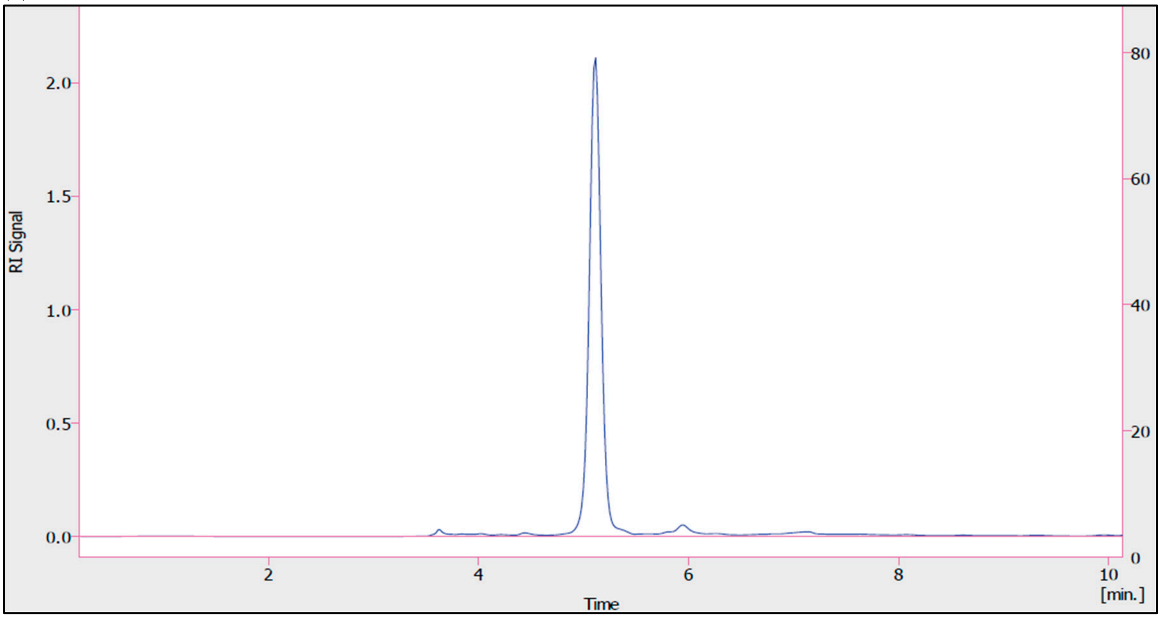
RI Signal
Time
[min.]

(d)

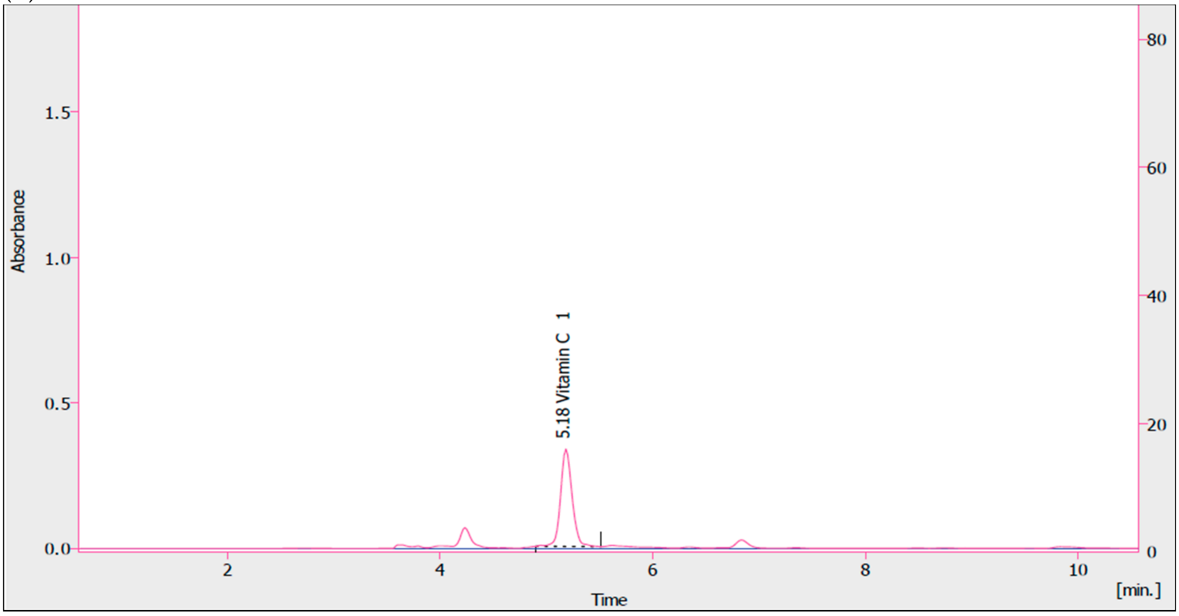

Figure S4. ICP-OES calibration curves for the determination of As, B, Ba, Ca, Cd, Co, Cu, Fe, K, Mg, Mn, Ni, Pb, Se, and Zn. The corresponding linear regression equations and correlation coefficients (r) are shown for each element.

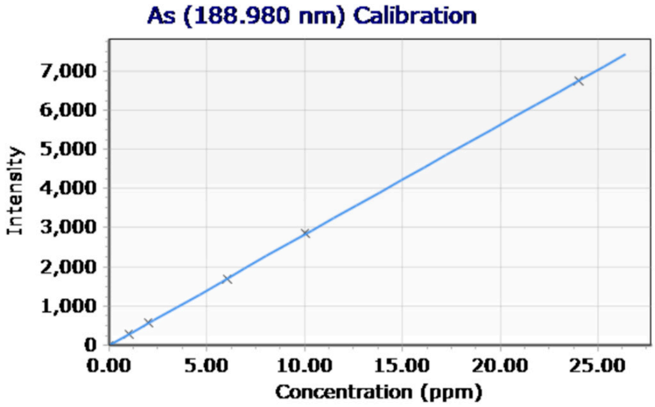

Intensity = 281.52011501 * Concentration + 7.59018000
Correlation coefficient: 0.99996

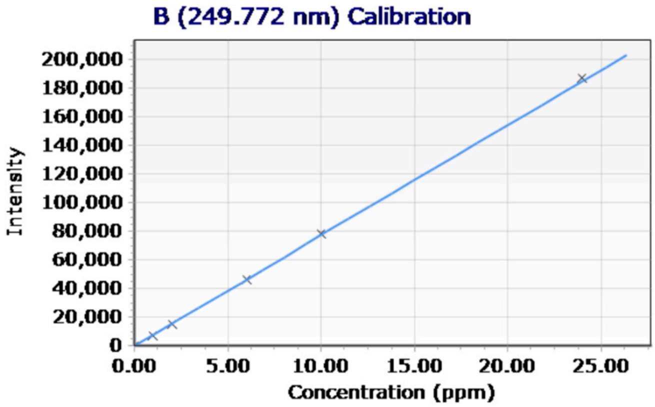

Intensity = 7692.98412734 * Concentration + 71.21997215

Correlation coefficient: 0.99998

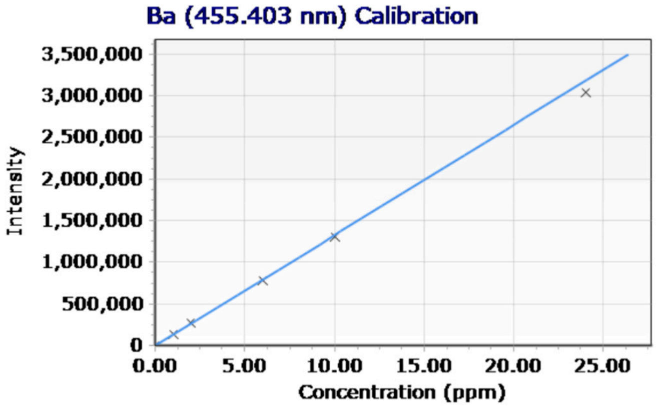

Intensity = 132591.77482406 * Concentration + 72.03706504
Correlation coefficient: 0.99990

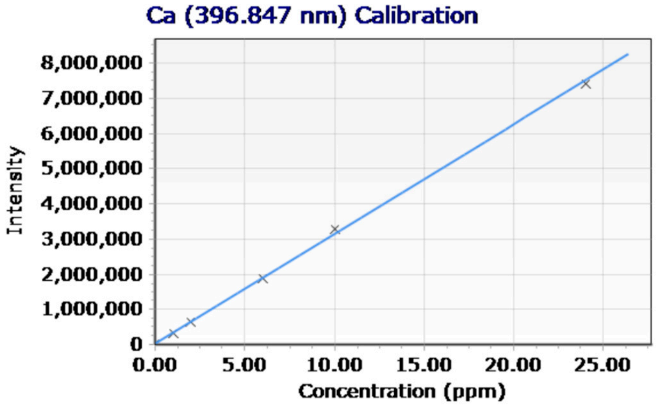

Intensity = 312821.70322775 * Concentration + 23285.06160748
Correlation coefficient: 0.99973

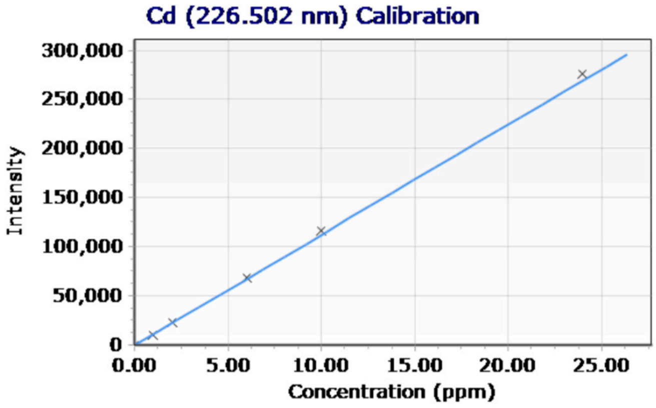

Intensity = 11223.28544141 * Concentration - 14.05856268
Correlation coefficient: 0.99997

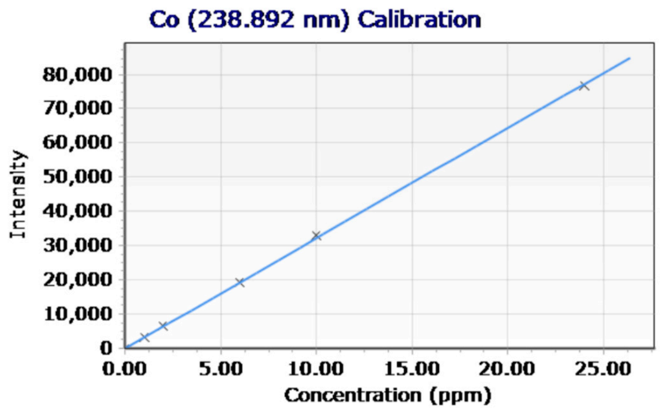

Intensity = 3216.71300554 * Concentration + 20.71368176
Correlation coefficient: 0.99991

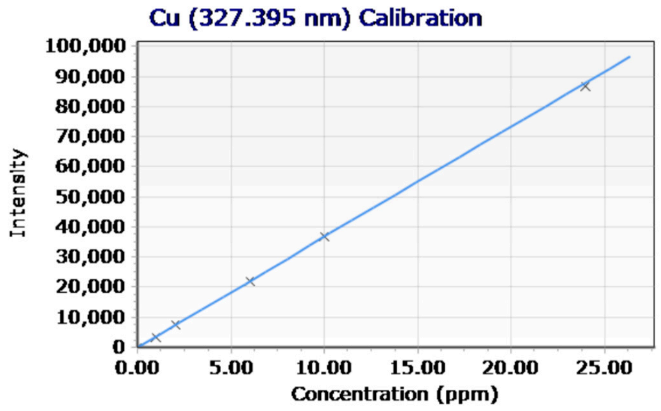

Intensity = 3665.29197714 * Concentration + 35.07211564
Correlation coefficient: 0.99996

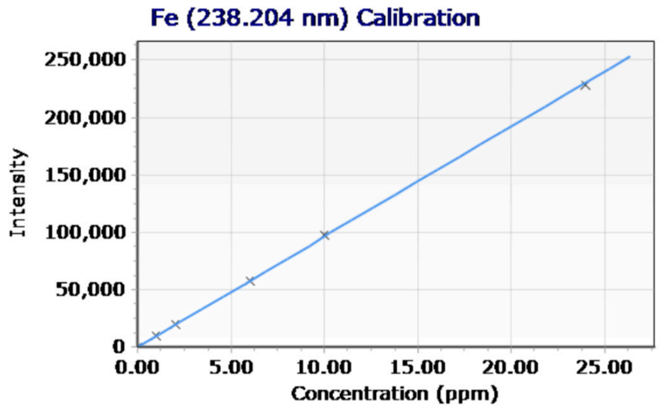

Intensity = 9608.96488144 * Concentration + 35.94102037
Correlation coefficient: 0.99994

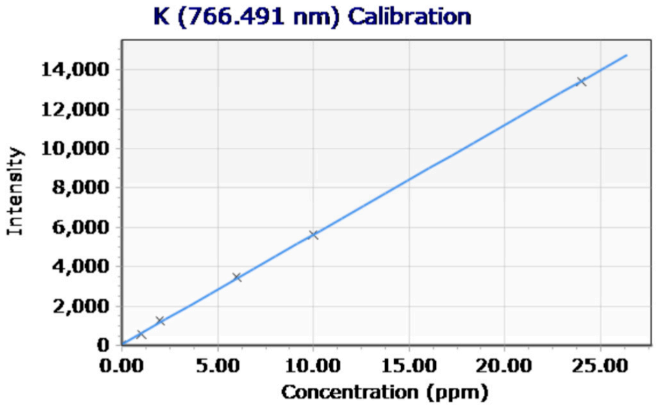

Intensity = 557.63499716 * Concentration + 64.92862971
Correlation coefficient: 0.99994

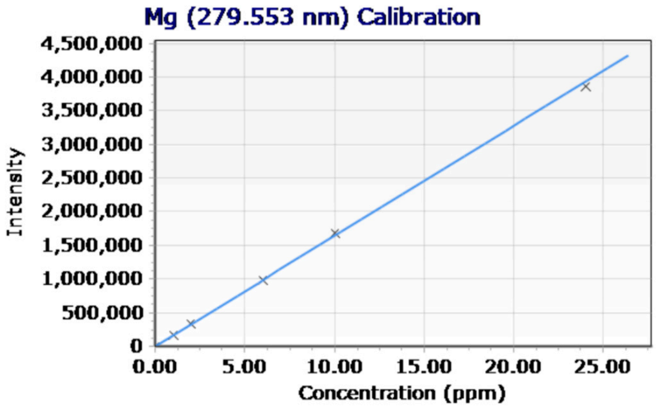

Intensity = 163725.37451233 * Concentration + 4481.30118594
Correlation coefficient: 0.99978

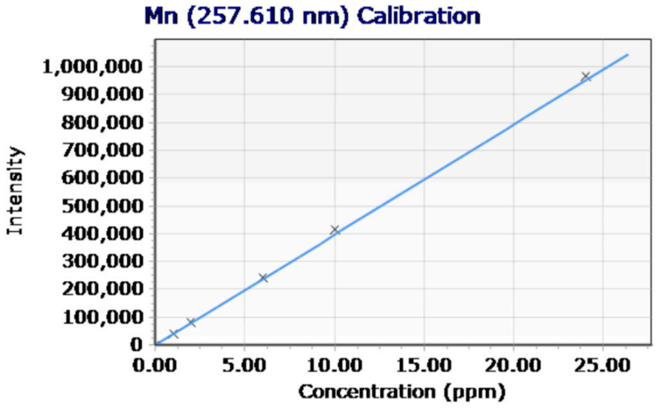

Intensity = 39642.43007756 * Concentration + 151.46427264
Correlation coefficient: 0.99994

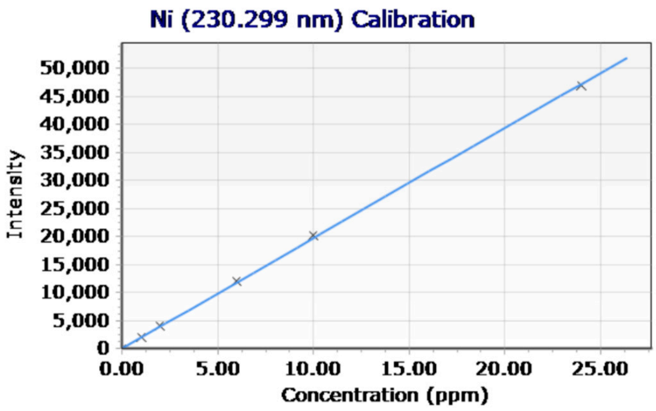

Intensity = 1967.38930665 * Concentration + 4.92455033
Correlation coefficient: 0.99988

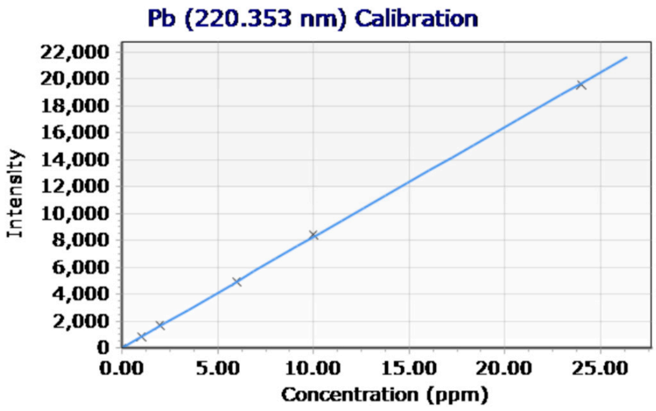

Intensity = 819.46838540 * Concentration + 23.93704054
Correlation coefficient: 0.99992

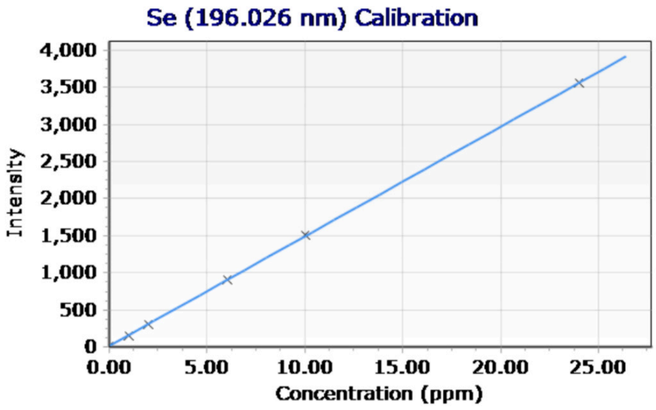

Intensity = 148.40738079 * Concentration + 8.79173419
Correlation coefficient: 0.99996

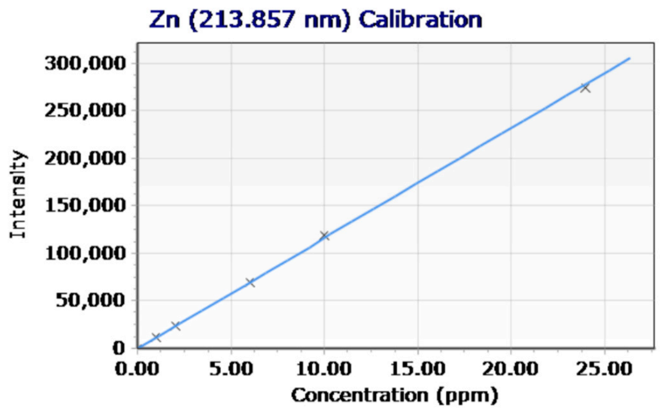

Intensity = 11586.84797978 * Concentration + 44.96789465
Correlation coefficient: 0.99988
